# Assessment of periodontal ligament visibility in third molars for threshold-based forensic age estimation: a cross-sectional study in a Turkish population

**DOI:** 10.1007/s00414-026-03753-w

**Published:** 2026-02-24

**Authors:** Mehmet Oguzhan Ergin, Kamile Erciyas, Sevim Samioglu Gunes, Aysun Baransel Isır

**Affiliations:** 1 Department of Periodontology, Faculty of Dentistry, Harran University, Sanliurfa, Turkey; 2 Department of Periodontology, Faculty of Dentistry, Gaziantep University, Gaziantep, Turkey; 3 Department of Forensic Medicine, Faculty of Medicine, Gaziantep University, Gaziantep, Turkey

**Keywords:** Forensic dentistry, Age estimation, Periodontal ligament, Third molars, Panoramic radiography

## Abstract

**Objectives:**

Forensic age estimation in living individuals is particularly challenging near legally relevant age thresholds after dental maturation is complete. This study aimed to evaluate the applicability of periodontal ligament (PDL) visibility staging in third molars as a post-maturation marker for threshold-based forensic age estimation, with specific focus on the 21-year age cut-off, in a Turkish population.

**Materials and methods:**

A retrospective analysis was conducted on 1,049 digital orthopantomograms (OPGs) of individuals aged 15–25 years. PDL visibility in maxillary and mandibular third molars was assessed according to the Olze et al. staging system. Associations between PDL stages, chronological age, sex, and tooth position were analyzed. Diagnostic performance for identifying individuals aged ≥ 21 years was evaluated using receiver operating characteristic (ROC) analysis.

**Results:**

Advanced PDL visibility stages were significantly associated with increasing age. Mandibular third molars provided the most informative staging patterns. ROC analysis demonstrated good discriminative accuracy for the 21-year threshold, with an area under the curve (AUC) of 0.79, indicating balanced sensitivity and specificity for threshold-based classification. Substantial intra- and inter-observer agreement confirmed methodological reliability.

**Conclusions:**

PDL visibility staging in third molars represents a reliable and reproducible complementary tool for forensic age estimation around the 21-year threshold in a Turkish population. Rather than exact age prediction, this method supports probabilistic decision-making in legally relevant age assessment and may contribute to a multimodal forensic age estimation framework.

## Introduction

 Forensic age estimation in living individuals is a fundamental component of legal and administrative decision-making, particularly in circumstances where the assessment of age thresholds such as 18 and 21 years carries significant legal consequences. These thresholds are decisive for issues including criminal responsibility, asylum and immigration procedures, and access to age-dependent civil rights. In such contexts, the absence of reliable identification documents necessitates the application of scientifically validated age estimation methods to support judicial and administrative authorities [1, 2].

Internationally accepted guidelines, such as those proposed by the Study Group on Forensic Age Diagnostics (AGFAD), recommend a multimodal approach to age estimation in living individuals. This approach integrates physical examination with radiographic assessment of skeletal and dental structures in order to improve diagnostic accuracy and minimize uncertainty [2]. Within this framework, dental methods occupy a central role, particularly during adolescence and early adulthood, as dental tissues are less susceptible to environmental, nutritional, and systemic influences than skeletal indicators [3].

Among dental approaches, assessment of third molar development has been widely applied in forensic age estimation due to the prolonged developmental timeline of these teeth. However, third molar root mineralization is frequently completed after the age of 18 years, most commonly during late adolescence and early adulthood, typically between 18 and 22 years, which limits the discriminatory capacity of traditional developmental staging methods when evaluating individuals near or beyond legal adulthood [4–6]. This limitation has prompted increasing interest in alternative dental markers that remain informative after the completion of tooth development.

From a biological standpoint, the periodontal ligament (PDL) represents a dynamic connective tissue that undergoes continuous remodeling throughout life. Age-related alterations in collagen fiber organization, cellular activity, and tissue turnover have been documented, reflecting both maturational and degenerative processes within the periodontal apparatus [7–9]. These biological changes may influence the radiographic appearance of the periodontal space, resulting in progressive alterations in the visibility of the PDL over time. Such changes provide a plausible biological rationale for the use of radiographic PDL visibility as an adjunct marker for forensic age estimation in living individuals once conventional developmental indicators have reached completion.

On this basis, several methods have been introduced to assess PDL visibility in third molars for forensic age estimation, including the staging systems proposed by Olze et al., Lucas et al., and Guo et al. [7, 8, 10]. Among these, the classification developed by Olze et al. evaluates the visibility of the periodontal ligament (PDL) along the entire root length and has been reported to demonstrate acceptable reproducibility and applicability in different populations [8]. More recently, methodological progress has been achieved through comparative radiological studies directly evaluating different PDL visibility staging systems for forensic age determination, thereby providing a more comprehensive framework for method selection and interpretation [11]. Nevertheless, population-specific validation remains essential, as variations in dental morphology, root configuration, and radiographic characteristics may influence the performance of PDL-based methods across different ethnic and demographic groups [10].

From an anatomical and radiographic perspective, mandibular third molars may provide more reliable information for PDL visibility assessment than maxillary third molars. Maxillary third molars are more susceptible to superimposition, maxillary sinus anatomy, and projection-related artifacts in panoramic radiography, which may obscure or exaggerate the periodontal ligament space. In contrast, mandibular third molars are less affected by these limitations and have therefore been preferentially used in PDL-based age estimation studies [7, 11, 13].

Therefore, the aim of the present study was to evaluate the applicability of the Olze et al. periodontal ligament visibility staging system for forensic age estimation in a Turkish population and to investigate its association with chronological age, tooth position, and sex.

## Materials and methods

### Study design and setting

This retrospective cross-sectional study was conducted using digital orthopantomograms (OPGs) retrieved from the archive of the Department of Oral and Maxillofacial Radiology, Gaziantep University Faculty of Dentistry. Radiographs had been acquired between January 2019 and May 2023 for routine diagnostic purposes. The study was designed in accordance with the principles outlined in the STROBE statement for observational studies [12].

## Study population and sample selection

A total archive of 6,000 digital OPGs constituted the initial data pool. From this archive, 1,049 OPGs belonging to individuals aged 15–25 years were included in the final study sample after application of predefined inclusion and exclusion criteria. Only one OPG per individual was considered.

Inclusion criteria comprised high-quality panoramic radiographs with adequate visualization of third molars, absence of positioning or exposure errors, and complete root development of third molars (corresponding to stage H according to Demirjian’s classification) [4]. Exclusion criteria included radiographs showing jaw pathologies, fractures, congenital or developmental anomalies affecting the jaws or teeth, previous orthodontic treatment, extensive restorations involving third molars, or insufficient image quality that hindered evaluation of the periodontal ligament (PDL).

Information regarding systemic diseases, congenital syndromes, or genetic disorders was assessed based on available clinical and medical records associated with the radiographic archive. Radiographs lacking accompanying clinical information or presenting conditions potentially influencing dental or skeletal development were excluded from the study.

The final sample consisted of 657 females (62.6%) and 392 males (37.4%), yielding a total of 1,049 individuals.

## Radiographic acquisition and image quality

All panoramic radiographs were obtained using the same panoramic imaging system (Planmeca ProMax^®^, Planmeca Oy, Helsinki, Finland) with standardized exposure parameters (60 kVp, 5 mA, 14.1 s) and a fixed magnification factor of 1.2. Digital images were stored in DICOM format and evaluated using dedicated viewing software that allowed image magnification and grayscale adjustment to optimize visualization of the periodontal ligament space.

## Assessment of periodontal ligament visibility

Radiographic visibility of the periodontal ligament was assessed in third molars (teeth 18, 28, 38, and 48) according to the staging system proposed by Olze et al. [8]. This classification evaluates the extent of PDL visibility along the entire root length and categorizes findings into four stages (Stages 0–3), reflecting progressive reduction in PDL visibility.

Each evaluable third molar was scored independently. In cases where more than one third molar was present, all eligible teeth were included in the analysis. Teeth that could not be reliably assessed due to superimposition, insufficient contrast, or other radiographic limitations were recorded as non-evaluable and excluded from stage-based analyses.

## Examiner calibration and reliability

All radiographic assessments were performed by a single experienced forensic dentist who was blinded to the chronological age and sex of the individuals. Prior to the main evaluation, a calibration session was conducted using a subset of radiographs not included in the study sample.

To assess intra-observer reliability, 100 randomly selected OPGs were re-evaluated after a three-month interval. Inter-observer reliability was assessed by a second independent examiner who evaluated the same set of radiographs. Agreement levels were calculated using Cohen’s kappa coefficient.

### Statistical analysis

Statistical analyses were performed using IBM SPSS Statistics for Windows (version 22.0; IBM Corp., Armonk, NY, USA). Descriptive statistics were calculated to summarize the distribution of sex, age, tooth position, and PDL visibility stages.

Associations between PDL visibility stages and chronological age, sex, and tooth position were evaluated using non-parametric statistical tests, including chi-square tests and Kruskal–Wallis tests, as appropriate. Spearman’s rank correlation coefficient was used to assess the relationship between PDL stages and chronological age due to the ordinal nature of the staging system. Intra- and inter-observer reliability were quantified using Cohen’s kappa coefficients. Statistical significance was set at *p* < 0.05. Diagnostic performance for identifying individuals aged ≥ 21 years was evaluated using receiver operating characteristic (ROC) analysis. For ROC analysis, classification was performed on an individual basis using the highest PDL visibility stage observed among the evaluable third molars of each subject.

## Results

### Sample characteristics

The final study sample consisted of 1,049 individuals aged 15–25 years, including 657 females (62.6%) and 392 males (37.4%). The mean chronological age was 21.02 ± 2.64 years, with a median age of 21 years (range: 15–25 years). Demographic characteristics of the study population are summarized in Table 1. The distribution of individuals across one-year age intervals is presented in Table 2.

**Table 1:** Demographic characteristics of the study sample (*N* = 1049)

Variable	Value
Age (years), mean ± SD	21.02 ± 2.64
Age (years), median (min–max)	21 (15–25)
Female, n (%)	657 (62.6%)
Male, n (%)	392 (37.4%)

Data are presented as mean ± standard deviation or median (minimum–maximum) for continuous variables and number (percentage) for categorical variables

**Table 2:** Distribution of individuals by chronological age and sex (*N* = 1049)

Age (years)	Total *n* (%)	Female (*n*)	Male (*n*)
15	18 (1.7)	9	9
16	29 (2.8)	18	11
17	67 (6.4)	36	31
18	87 (8.3)	51	36
19	92 (8.8)	55	37
20	138 (13.2)	94	44
21	118 (11.2)	79	39
22	141 (13.4)	88	53
23	139 (13.3)	84	55
24	130 (12.4)	88	42
25	90 (8.6)	55	35
Total	1049 (100.0)	657	392

Values are presented as number (percentage of the total study population)

### Distribution of periodontal ligament visibility stages

The distribution of periodontal ligament (PDL) visibility stages according to the Olze classification is shown in Table 3. Stage 3 was the most frequently observed category (33.7%), followed by Stage 1 (28.7%), Stage 2 (19.2%), and Stage 0 (18.4%). It should be noted that the distribution of PDL visibility stages shown in Table 3 partly reflects the underlying age composition of the study sample, with older age groups being more heavily represented.

**Table 3:** Distribution of PDL visibility stages (Olze classification)

PDL Stage	*n*	%
Stage 0	193	18.4
Stage 1	301	28.7
Stage 2	201	19.2
Stage 3	354	33.7
Total	1049	100.0

PDL visibility stages were assessed according to the Olze et al. classification (Stages 0–3). Percentages refer to the total number of evaluable observations

### Association between PDL visibility and chronological age

A statistically significant positive association was observed between chronological age and PDL visibility stage. Spearman’s rank correlation analysis demonstrated a moderate correlation between increasing age and higher PDL stages (ρ = 0.565, *p* < 0.001).

### Distribution of PDL visibility stages by tooth position

PDL visibility stages differed significantly according to third molar position (χ² = 189.9, df = 9, *p* < 0.001). Maxillary third molars (teeth 18 and 28) demonstrated a higher prevalence of advanced PDL stages, whereas mandibular third molars (teeth 38 and 48) showed a broader distribution across intermediate stages. Detailed stage distributions by tooth position are presented in Table 4.

**Table 4:** Distribution of PDL visibility stages by third molar position

Tooth	Stage 0	Stage 1	Stage 2	Stage 3	Total
18	6	11	11	85	113
28	10	15	11	75	111
38	95	150	100	84	429
48	82	125	79	110	396
Total	193	301	201	354	1049

Values are presented as number (percentage within each tooth group). Differences in stage distribution across tooth positions were evaluated using the chi-square test

In accordance with the minimum age concept, the minimum observed chronological age for each PDL visibility stage was recorded. In the present cohort, the minimum ages for Stages 0, 1, 2, and 3 were 15, 15, 15, and 17 years, respectively. These values represent descriptive minima within the study sample and should not be interpreted as diagnostic cut-offs.

### Sex-based differences in pdl visibility

A significant association was identified between sex and PDL visibility stages (χ² = 10.88, df = 3, *p* = 0.012). Male individuals exhibited a higher prevalence of advanced PDL stages (Stage 3), while females more frequently presented Stage 1. The distribution of PDL stages by sex is shown in Table 5. This distribution should be interpreted in light of the age structure of the study population, as the observed sex-based differences partly reflect the unequal representation of individuals across age groups.

**Table 5:** Distribution of PDL visibility stages by sex

Sex	Stage 0	Stage 1	Stage 2	Stage 3	Total
Female	121	205	132	199	657
Male	72	96	69	155	392

Values are presented as number (percentage within sex groups). Associations were assessed using the chi-square test

### Examiner reliability

Intra-observer agreement was almost perfect (Cohen’s κ = 0.93; 95% CI: 0.86–0.99; quadratic weighted κ = 0.97; 95% CI: 0.94–0.99; *n* = 100). Inter-observer agreement was substantial to almost perfect (Cohen’s κ = 0.86; 95% CI: 0.78–0.93; quadratic weighted κ = 0.95; 95% CI: 0.91–0.98; *n* = 100).

### Diagnostic performance for the 21-year age threshold

Binary analysis using a 21-year age threshold demonstrated that PDL visibility stages 2–3 yielded a sensitivity of 72.7% and a specificity of 75.4% for identifying individuals aged 21 years and older. The positive predictive value was 80.9%, and the negative predictive value was 65.8%. Diagnostic performance measures are summarized in Table 6.

**Table 6:** Diagnostic performance of PDL visibility for the 21-year threshold

Parameter	Value
Sensitivity	72.7%
Specificity	75.4%
Positive Predictive Value (PPV)	80.9%
Negative Predictive Value (NPV)	65.8%

PDL visibility stages 2–3 were considered positive for age ≥ 21 years, whereas stages 0–1 were considered negative. Chronological age was used as the reference standard

### Receiver operating characteristic (ROC) analysis

**Fig. 1:**
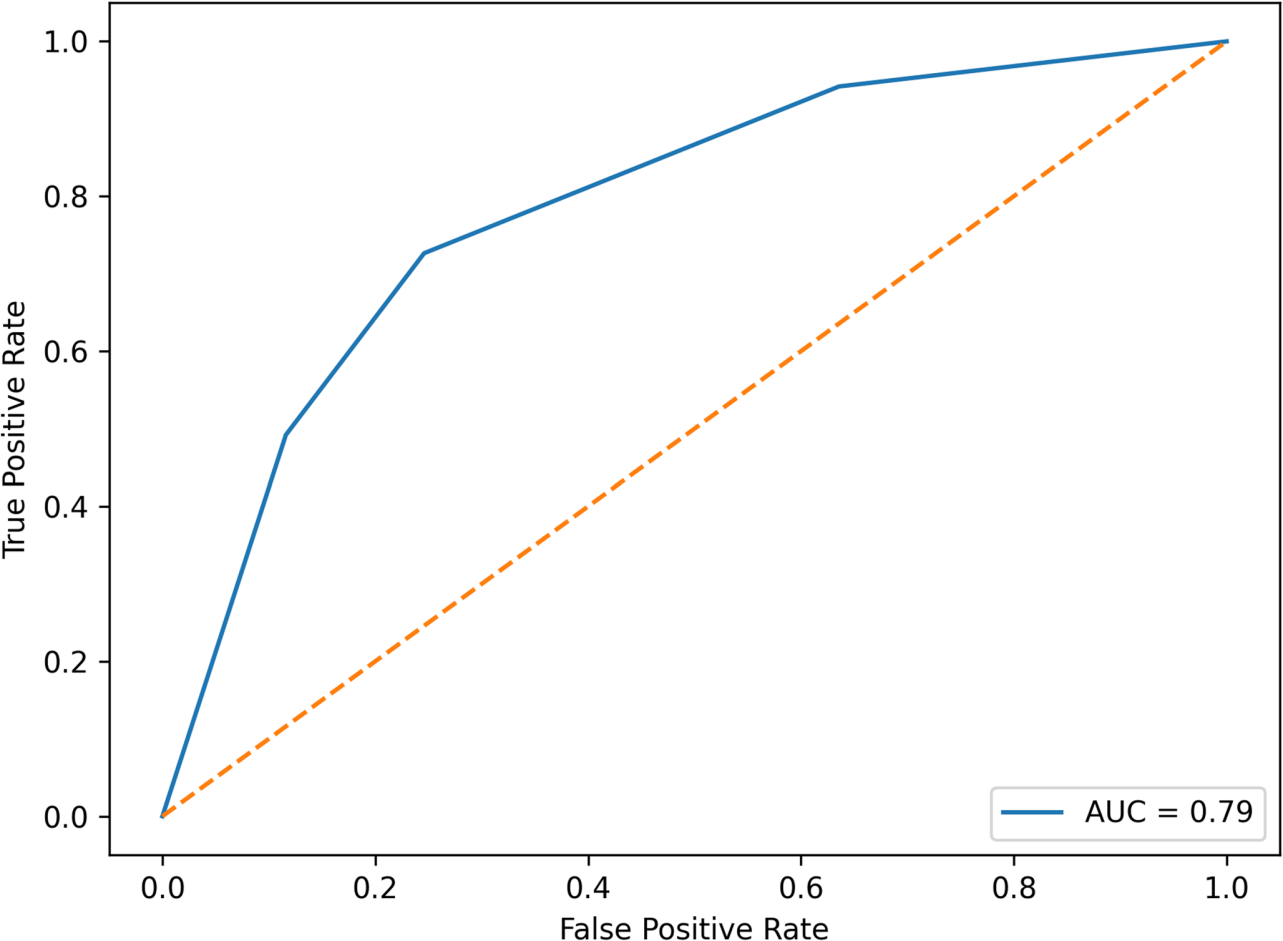
Receiver operating characteristic (ROC) curve illustrating the discriminatory ability of periodontal ligament (PDL) visibility stages (Olze classification) for the 21-year age threshold. Chronological age (≥ 21 vs. < 21 years) was used as the reference standard. The area under the curve (AUC) was 0.79, indicating good overall accuracy

Receiver operating characteristic (ROC) analysis was conducted to assess the discriminatory ability of PDL visibility stages for the 21-year age threshold. The analysis yielded an area under the curve (AUC) of 0.79, indicating good overall accuracy. The ROC curve is shown in Fig. 1.

## Discussion

The present study demonstrated that periodontal ligament (PDL) visibility staging provides good discriminatory performance for identifying individuals aged 21 years and older in a Turkish population. Using a threshold-based approach, dichotomization of PDL visibility stages (stages 2–3 versus stages 0–1) yielded a sensitivity of 72.7% and a specificity of 75.4%, with a positive predictive value of 80.9% and a negative predictive value of 65.8% for the 21-year age cut-off. Receiver operating characteristic (ROC) analysis further confirmed good overall diagnostic accuracy, with an area under the curve (AUC) of 0.79. These findings indicate that PDL visibility staging can support probabilistic decision-making in legally relevant age assessment rather than exact chronological age prediction.

The diagnostic performance observed in the present study is in line with earlier investigations evaluating PDL visibility as a post-developmental dental marker. Olze et al. were the first to report that advanced PDL visibility stages in lower third molars are associated with increasing chronological age and may be used to support forensic age estimation beyond the completion of dental development [8]. Subsequent studies conducted in different populations have confirmed the applicability and reproducibility of the Olze classification, although the reported diagnostic performance has shown some variability depending on population characteristics, tooth position, and the applied age threshold [13–17]. In a recent comparative study, Timme et al. directly evaluated three different PDL visibility staging systems and demonstrated that the Olze method provides diagnostic performance comparable to alternative scales, with robust observer agreement [11]. In this context, the AUC value of 0.79 observed in the present Turkish sample is fully consistent with previously reported findings and supports the external validity of this approach.

With regard to sensitivity and specificity, the balance observed in the present study aligns well with previous threshold-based dental age estimation research. Several studies focusing on legally relevant age cut-offs, particularly the 18-year threshold, have reported similar trade-offs when PDL visibility was used as a diagnostic indicator [17, 18]. Although these studies addressed a younger age limit, they consistently emphasized that specificity tends to exceed sensitivity when PDL visibility is applied, which is a desirable characteristic in forensic contexts. As highlighted by Bassed et al. and Focardi et al., high specificity and positive predictive value are of particular importance to minimize the ethical and legal consequences of incorrectly classifying a minor as an adult [9, 10]. In this respect, the specificity of 75.4% and PPV of 80.9% observed in the present study represent meaningful and defensible performance characteristics for forensic application at the 21-year threshold.

A major contribution of the present study is its explicit focus on the 21-year age threshold, which has received comparatively limited attention in dental age estimation research. While third molar mineralization and eruption remain informative during late adolescence, their discriminatory capacity decreases substantially in the early twenties once root development is complete [4–6]. This limitation has led to increasing interest in post-developmental indicators that reflect age-related tissue changes rather than genetically programmed growth processes. In contrast to developmental markers, PDL visibility reflects ongoing biological remodeling and adaptive changes within the periodontal tissues, allowing age-related information to be extracted beyond dental maturation.

The biological plausibility of PDL visibility as an age-related marker further supports its forensic applicability. The periodontal ligament is a dynamic connective tissue that undergoes continuous remodeling throughout life. Age-associated changes in cellularity, collagen fiber organization, and mineralization at the bone–ligament interface have been well documented and are known to influence the radiographic appearance of the periodontal ligament space [7, 9]. These microstructural alterations provide a biological explanation for the progressive reduction in PDL visibility observed with increasing age. Unlike conventional dental developmental markers, which plateau after maturation, PDL visibility retains age dependency into young adulthood, thereby offering complementary information for age estimation in post-maturational individuals [8, 14].

Sex-related differences in PDL visibility were observed in the present study, with males showing a higher prevalence of advanced PDL stages. Similar findings have been reported in previous investigations and may be attributable to sex-related differences in craniofacial morphology, bone density, and periodontal tissue remodeling patterns [11, 17]. However, as the primary aim of the present study was to evaluate a unified threshold-based approach applicable to routine forensic practice, sex-specific diagnostic models were not developed. The observed differences should therefore be interpreted descriptively and in the context of the underlying age distribution of the study population rather than as evidence of distinct sex-specific cut-offs.

The reliability analysis demonstrated substantial to almost perfect intra- and inter-observer agreement, confirming that PDL visibility staging can be applied consistently when examiners are appropriately trained and calibrated. These agreement values are comparable to, or higher than, those reported in previous PDL visibility studies [8, 11, 14]. High reproducibility is a fundamental prerequisite for forensic applicability, as expert assessments must be transparent, repeatable, and defensible under legal scrutiny.

Several limitations should be acknowledged. The retrospective, single-center design may limit generalizability, and the age distribution of the study sample was intentionally concentrated around the legally relevant threshold of 21 years. While this focus may influence descriptive stage frequencies and prevalence-dependent measures such as predictive values, discrimination-based metrics derived from ROC analysis, including the AUC, are considered largely independent of prevalence and therefore provide a robust assessment of threshold-based performance [11, 12]. In addition, panoramic radiography is inherently subject to distortion, magnification, and focal trough limitations, which may affect PDL visibility assessment in individual cases [11]. Consequently, PDL visibility staging should not be interpreted as a standalone determinant of chronological age.

In accordance with internationally accepted forensic guidelines, PDL visibility should be integrated into a multimodal age estimation framework alongside other dental and skeletal indicators, such as third molar development and clavicular epiphyseal assessment [1, 2, 9]. Within such comprehensive protocols, the present study provides robust population-specific evidence supporting the value of PDL visibility staging as a complementary tool for threshold-based forensic age assessment in Turkish adolescents and young adults.

## Conclusion

Radiographic assessment of periodontal ligament (PDL) visibility in third molars provides a reliable post-maturation marker for forensic age estimation in a Turkish population. A threshold-based approach focusing on the 21-year cut-off demonstrated good discriminative performance, as supported by ROC analysis, emphasizing its value for legally relevant age assessment rather than exact age prediction. Mandibular third molars yielded the most informative patterns, with substantial observer agreement confirming methodological reliability. PDL visibility staging may therefore serve as a useful complementary component within a multimodal forensic age estimation framework.

PDL visibility stages 2–3 were considered positive for age ≥ 21 years, whereas stages 0–1 were considered negative. Chronological age was used as the reference standard.

## Data Availability

The data that support the findings of this study are available from the corresponding author upon reasonable request.
